# Pentadecanoic Acid, an Odd-Chain Fatty Acid, Suppresses the Stemness of MCF-7/SC Human Breast Cancer Stem-Like Cells through JAK2/STAT3 Signaling

**DOI:** 10.3390/nu12061663

**Published:** 2020-06-03

**Authors:** Ngoc Bao To, Yen Thi-Kim Nguyen, Jeong Yong Moon, Meran Keshawa Ediriweera, Somi Kim Cho

**Affiliations:** 1 Interdisciplinary Graduate Program in Advanced Convergence Technology and Science, Jeju National University, Jeju 63243, Korea; tobaongoc.hcmus@gmail.com (N.B.T.); ntkyen.hcmus@gmail.com (Y.T.-K.N.); 2 Subtropical/Tropical Organism Gene Bank, Jeju National University, Jeju 63243, Korea; owenmjy@jejunu.ac.kr (J.Y.M.); or mk.ediriweera@gmail.com (M.K.E.)

**Keywords:** breast cancer, odd-chain fatty acids, pentadecanoic acid, JAK2/STAT3 signaling, cancer stem cells, apoptosis

## Abstract

Saturated fatty acids possess few health benefits compared to unsaturated fatty acids. However, increasing experimental evidence demonstrates the nutritionally beneficial role of odd-chain saturated fatty acids in human health. In this study, the anti-cancer effects of pentadecanoic acid were evaluated in human breast carcinoma MCF-7/stem-like cells (SC), a cell line with greater mobility, invasiveness, and cancer stem cell properties compared to the parental MCF-7 cells. Pentadecanoic acid exerted selective cytotoxic effects in MCF-7/SC compared to in the parental cells. Moreover, pentadecanoic acid reduced the stemness of MCF-7/SC and suppressed the migratory and invasive ability of MCF-7/SC as evidenced by the results of flow cytometry, a mammosphere formation assay, an aldehyde dehydrogenase activity assay, and Western blot experiments conducted to analyze the expression of cancer stem cell markers—CD44, β-catenin, MDR1, and MRP1—and epithelial–mesenchymal transition (EMT) markers—snail, slug, MMP9, and MMP2. In addition, pentadecanoic acid suppressed interleukin-6 (IL-6)-induced JAK2/STAT3 signaling, induced cell cycle arrest at the sub-G1 phase, and promoted caspase-dependent apoptosis in MCF-7/SC. These findings indicate that pentadecanoic acid can serve as a novel JAK2/STAT3 signaling inhibitor in breast cancer cells and suggest the beneficial effects of pentadecanoic acid-rich food intake during breast cancer treatments.

## 1. Introduction

According to the latest cancer statistics, breast cancer ranks as the leading cause of mortality among women [1]. Currently, surgery, chemotherapy, hormone therapy, and radiotherapy are widely used to treat breast cancer patients [2]. Convincing evidence demonstrates that the presence of a small subpopulation cells, called breast cancer stem cells (BCSCs), play a pivotal role in breast cancer therapy resistance [3,4], metastasis, and tumor recurrence [5]. BCSCs are known to express higher levels of drug efflux transporters such as P-glycoprotein (P-gp/ABCB1) and multidrug resistance-associated protein 1 (MRP1/ABCC1) [6]. CD44, CD24, CD133, EpCAM, CD166, CD47, aldehyde dehydrogenases (ALDH), and ABCG2 have been identified as key BCSC markers [7,8,9,10]. A number of natural compounds have been reported to exert CSC inhibitory properties through targeting multiple cancer signaling pathways [8,11]. Therefore, natural compounds can be identified as new chemotherapeutic agents, with special attention focused on their efficacy to target BCSCs [12,13,14].

It has been reported that a number of intracellular signaling pathways are frequently dysregulated in BCSCs [8]. The JAK2/STAT3 signaling pathway has been reported to control BCSC survival, growth, transition, and drug resistance through promoting EMT [15,16]. JAK2 is a non-receptor tyrosine kinase that can associate with the cytoplasmic domains of several peptides, cytokines, and growth factors. The transcription factor STAT3 is involved in a number of vital biological events. Notably, the aberrant regulation of STAT3 is reported in a range of human cancers including breast cancer, making it a potential therapeutic target [17]. According to several pre-clinical investigations, activated STAT3 can play a regulatory role in breast cancer therapy resistance and tumorigenesis. It has been reported that silencing STAT3 can induce apoptosis through the inhibition of the expression of several oncogenic proteins in vitro and in vivo [18,19,20]. Considering the regulatory role of STAT3 in breast cancer drug resistance and tumorigenesis, the exploration of new STAT3 inhibitors will assist the development new STAT3-targeted anti-cancer therapies for breast cancer.

Saturated fatty acids are found in both animal and plant tissues and important as the substrates for metabolic energy, membrane biogenesis, and signaling components [21,22]. Besides being classified according to their saturation, saturated fatty acids can also be classified into two main groups depending on the number of carbon atoms: odd and even-chain fatty acids [22]. Among them, even-chain fatty acids are abundantly found in the human plasma (99%). However, a small amount of odd-chain fatty acids (OCFAs) is also detected in the human plasma. Some plants, ruminant milk fat, and fish oils are reported to contain high amounts of OCFAs: pentadecanoic acid (C15:0) and heptadecanoic acid (C17:0) [23,24]. To date, pentadecanoic acid (C15:0) and heptadecanoic acid (C17:0) have been widely utilized as internal standards in gas chromatography experiments, dairy food intake bio-markers, and bio-markers for coronary heart diseases and type II diabetes mellitus [25,26,27,28]. Interestingly, a recent study demonstrates that heptadecanoic acid (C17:0) can exert anti-cancer effects in gefitinib-resistant non-small cell lung carcinoma (NSCLC) cells, emphasizing the efficacy of OCFAs in targeting human lung cancer cells [29]. Nevertheless, investigations assessing the anti-cancer effects of odd-chain fatty acids (C15:0 and C17:0) are extremely limited, and no studies have evaluated the in vitro anti-cancer effects of pentadecanoic acid (C15:0).

In this study, we explore the anti-cancer activity of pentadecanoic acid and the underlying molecular mechanisms responsible for this activity in MCF-7/SC human breast cancer stem-like cells. We demonstrate that pentadecanoic acid suppresses the stemness and induces apoptosis through targeting the JAK2/STAT3 signaling pathway. These studies will provide experimental evidence to understand the molecular mechanisms underlying the anti-cancer effects of OCFAs and a rationale to use OCFAs in nutrition therapy for breast cancer patients.

## 2. Materials and Methods

### 2.1. Cell Lines and Cell Culture

An MCF-7/SC population, which was established from human estrogen receptor-positive (ER+) breast carcinoma cells (MCF-7) by sorting with CD44^+^/CD24^−^ antibodies, was used in the present investigation [30,31]. MCF-10A cells were purchased from the American Type Culture Collection (ATCC, Rockville, MD, USA). MCF-7 cells and MCF-7/SC were cultured in DMEM (10% FBS, 100 U/mL Of penicillin and 100 µg/mL of streptomycin) and RPMI-1640 (10% FBS, 100 U/mL of penicillin and 100 µg/mL of streptomycin) media, respectively. Normal MCF-10A cells were cultured with the ATCC-recommended culture medium and cell culture conditions.

### 2.2. Cell Viability Assay

The MTT assay was conducted as the cell viability assay in the present study. Briefly, MCF-7/SC (2000 cells/well) were seeded in 96-well plates and incubated for 24 h. Following incubation, the MCF-7/SC were exposed to different fatty acids (C15:0, C17:0, C18:1, and C18:2) for 48 h. Prior to the assay, fatty acids were dissolved in ethanol and filtered. After 48 h, cells were incubated with the MTT solution (100 μL of 1 mg/mL) for 2 h at 37 °C. One hundred and fifty microliters of dimethyl sulfoxide (DMSO) was then added to each well, and the plates were shaken for 30 min at room temperature. Absorbance was recorded at 570 nm using a micro-plate reader, and cell viability was calculated as described in our recent study [32].

### 2.3. Wound Healing Assay

MCF-7/SC (1.5 × 10^5^ cells/well) were seeded in 6-well plates and incubated until the cells reached 95% confluency. Then, uniform scratches (width, ~1 mm) were made in the cell monolayers using a sterile pipette tip, and the cells were washed with PBS to remove detached cells, followed by culturing in RPMI-1640 with or without pentadecanoic acid. Following 48 h of incubation, wound areas were captured using an inverted phase-contrast microscope.

### 2.4. Cell Invasion Assay

Trans-wells (24-well plates) were used in the cell invasion assay. Prior to the assay, the upper chambers of the trans-wells were coated with 1% Matrigel. The coated upper chambers received MCF-7/SC (1.5 × 10^5^ per Transwell) cells supplemented with or without pentadecanoic acid, while the lower chambers received RPMI-1640 supplemented with 10% FBS. Following 48 h of incubation, 4% paraformaldehyde and methanol were used to fix cells. The fixed cells were stained (2% crystal violet) and observed under a phase-contrast microscope.

### 2.5. Flow Cytometry

MCF-7/SC (3 × 10^4^ cells) were seeded in 60 mm dishes for 24 h and treated with different concentrations of pentadecanoic acid for 48 h. Following 48 h of incubation, the expression of breast cancer stem cell surface markers, CD24 and CD44, was analyzed using fluorescence-activated cell sorting (FACS) as described in our recent study [33]. CD24 (PE-conjugated anti-human CD24 antibody) and CD44 (FITC-conjugated anti-human CD44 antibody) were purchased from BD Pharmingen, San Diego, CA, USA.

### 2.6. Aldefluor Assay

ALDH enzyme activity was determined using the ALDEFLUOR Kit (Stemcell Technologies, Vancouver, BC, Canada) according to the manufacturer’s instructions. To carry out the assay, MCF-7/SC (3 × 10^4^ cells/mL) were seeded in cell culture dishes and incubated for 24 h. After 24 h of incubation, the cells were exposed to pentadecanoic acid (0, 50, 100, 150, or 200 μM) for 48 h and analyzed using flow cytometry. Dimethylaminobenzaldehyde (DEAB), an ALDH inhibitor, was used as the negative control.

### 2.7. Mammosphere Formation Assay

MCF-7/SC (2 × 10^4^ cells/mL) were cultured in ultralow-attachment cell culture dishes containing MammoCult Human Medium (Stemcell Technologies, Vancouver, Canada)) supplemented with or without pentadecanoic acid (0, 50, 100, 150, or 200 μM). Following 10 days of incubation, mammospheres (>60 μm) were captured using a phase-contrast microscope.

### 2.8. Cell Cycle Analysis

MCF-7/SC (3 × 10^4^ cells) were seeded in 60 mm cell culture dishes and incubated for 24 h. Following incubation, the cells were exposed to pentadecanoic acid (0, 50, 100, 150, or 200 μM) for 48 h. Cell cycle analysis was carried out as described in our recent study [33].

### 2.9. Annexin V/PI Staining

MCF-7/SC (3 × 10^4^ cells) were seeded in cell culture dishes and incubated for 24 h. Following 48 h of incubation, the cells were exposed to pentadecanoic acid (0, 50, 100, 150, or 200 μM) for 48 h. Apoptosis in MCF-7/SC was detected using the annexin *V-FITC Apoptosis Detection Kit* following the supplier’s instructions.

### 2.10. Western Blotting

MCF-7/SC were exposed to different concentrations of pentadecanoic acid for 48 h. Following incubation, the cells were lysed using the radioimmunoprecipitation assay (RIPA) buffer. After quantifying the proteins in the cell lysates, they were separated using SDS-PAGE. The separated proteins were transferred to a PVDF membrane, and the membranes were blocked with skim milk, followed by incubation with different primary antibodies. Except GAPDH, the primary antibodies were diluted a thousand fold in skim milk. All the primary antibodies were purchased from Cell Signaling Technology (Beverly, MA, USA). Secondary antibodies, anti-rabbit and anti-mouse immunoglobulin G (IgG) (Vector Laboratories, Burlingame, CA, USA), were diluted five thousand fold. The BS ECL Plus Kit (Biosesang, Seongnam, South Korea) was used to develop the proteins.

### 2.11. Reactive Oxygen Species (ROS) Generation Analysis

Briefly, MCF-7/SC (3 × 10^4^) were seeded in cell culture dishes and incubated for 48 h. After incubation, the cells were stained with 2′,7′-dichlorofluorescein diacetate (H2DCFDA), a fluorescent probe used to detect ROS, for 15 min. Following 15 min of incubation, the stained cells were washed with PBS and analyzed by flow cytometry.

### 2.12. Statistical Analysis

The GraphPad Prism 7.0 software (La Jolla, CA, USA) was used for statistical analysis in the present study. The data are expressed as the mean ± SD of at least three independent experiments and statistically analyzed using the Student’s t-test. *p* < 0.05 (*) was considered as significant.

## 3. Results

### 3.1. MCF-7/SC Displayed Higher Stem Cell Characteristics Compared to the Parental MCF-7 Cells

**Figure 1:**
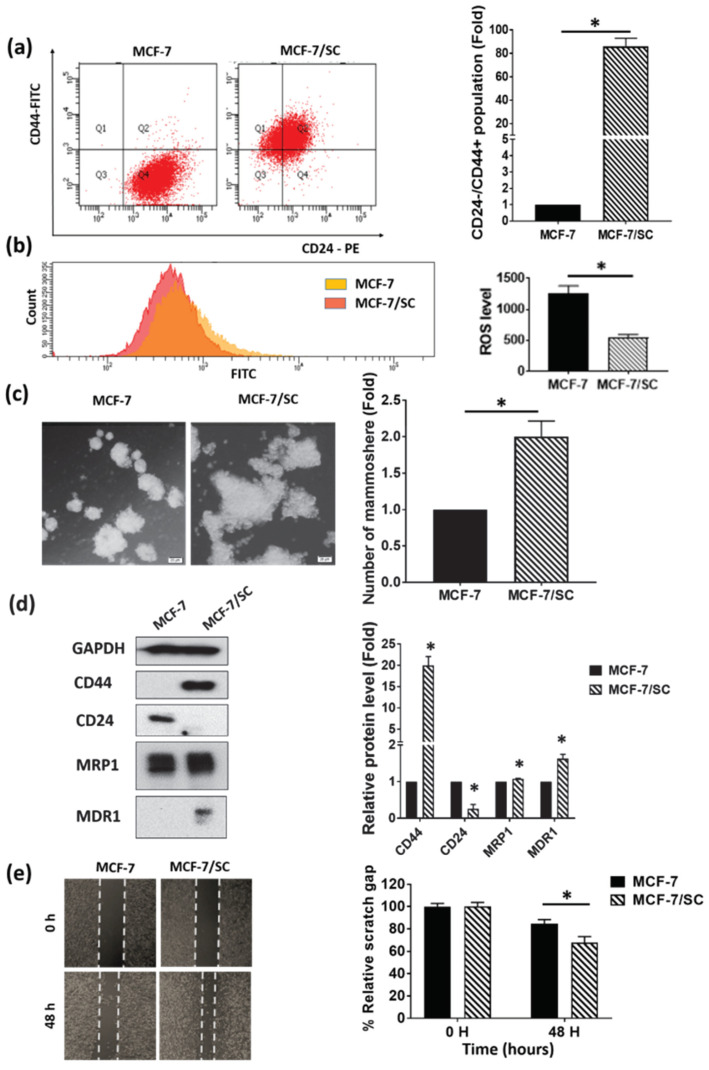
MCF-7/SC exhibit more prominent cancer stem cell characteristics than the parental MCF-7 cells. (**a**) Fluorescence-activated cell sorting (FACS) analysis of the CD44^+^/CD24^−^ cell population in MCF-7/SC and MCF-7 cells. (**b**) Measurement of the ROS levels in MCF-7/SC and MCF-7 cells. (**c**) Comparison of the mammosphere formation ability of MCF-7/SC and MCF-7 cells cultured in the MammoCult Human Medium for 10 days. Magnification 100×. (**d**) Analysis of the expression of cancer stem cell markers in MCF-7/SC and MCF-7 cells by Western blotting. GAPDH was used as a loading control. (**e**) Migratory potential of MCF-7/SC and MCF-7 cells as assessed by the wound healing assay. Data are shown as the mean ± standard deviation of three biologically independent experiments. * *p* < 0.05 vs. control.

The FACS technique was employed to compare the expression of cell surface markers (CD44^+^/CD24^-^) in MCF-7/SC and parental MCF-7 cells. As shown in Figure 1a, MCF-7/SC displayed an enriched CD44^+^/CD24^−^ cell population compared to MCF-7 cells, indicating the characteristics of cancer stem cells. We then compared the reactive oxygen species (ROS) levels in MCF-7/SC and MCF-7 cells. As shown in Figure 1b, the MCF-7/SC were found to contain lower ROS levels than the MCF-7 cells, which is a common feature of cancer stem cells [34]. Moreover, the MCF-7/SC displayed an increased ability to form mammospheres (Figure 1c). In addition, according to the results of Western blot experiments, MCF-7/SC were found to possess higher levels of cancer stem cell markers such as CD44, MRP1, and MDR1 and lower levels of CD24 compared with MCF-7 cells (Figure 1d). Furthermore, MCF-7/SC exhibited enhanced migratory potential compared to MCF-7 cells (Figure 1e). Altogether, these results clearly demonstrate that MCF-7/SC can be considered as stem-like cells that possess an enriched CSC population.

### 3.2. Pentadecanoic Acid Exerted Significant Cytotoxicity in MCF-7/SC

**Figure 2:**
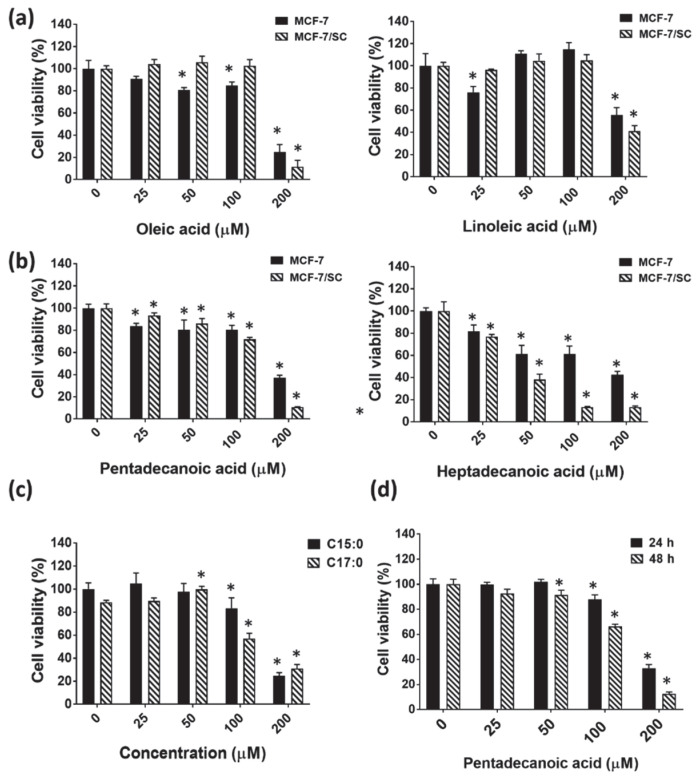
Pentadecanoic acid exerts cytotoxicity in MCF-7/SC and MCF-7 cells. Comparison of cytotoxic effects in MCF-7/SC and MCF-7 cells following 48 h of incubation. (**a**) Unsaturated fatty acids: oleic acid (C18:1) and linoleic acid (C18:2). (**b**) Odd-chain fatty acids: pentadecanoic acid (C15:0) and heptadecanoic acid (C17:0). (**c**) Effects of pentadecanoic acid and heptadecanoic acid on MCF-10A cell proliferation after 48 h of incubation. (**d**) Time-dependent (24 and 48 h) cytotoxic effects of pentadecanoic acid in MCF-7/SC. Data are shown as the mean ± standard deviation of three biologically independent experiments. * *p* < 0.05 vs. control.

In previous investigations, oleic acid (C18:1), an unsaturated fatty acid, has been shown to exert anti-cancer effects in esophageal [35], breast [36], and tongue [37] cancer cells. In addition, linoleic acid (C18:2), another example for an unsaturated fatty acid, has been shown to suppress the proliferation of colorectal cancer cells [38,39]. Considering the available reports on the anti-cancer effects of unsaturated fatty acids, we decided to include oleic acid and linoleic acid in cytotoxicity assays together with odd-chain fatty acids (OCFAs). The cytotoxic effects of the unsaturated fatty acids oleic acid and linoleic acid and odd-chain fatty acids pentadecanoic acid (C15:0) and heptadecanoic acid (C17:0) were evaluated by the MTT assay in MCF-7/SC and MCF-7 cells following 48 h of exposure. As shown in Figure 2a, the unsaturated fatty acids (oleic acid and linoleic acid) exhibited relatively lower cytotoxicity in both MCF-7/SC and MCF-7 cells compared to the saturated fatty acids (pentadecanoic acid and heptadecanoic acid). Heptadecanoic acid exerted higher cytotoxicity in MCF-7/SC compared to pentadecanoic acid in MCF-7/SC with IC_50_ values for heptadecanoic acid of 41.94 ± 4.06 µM and for pentadecanoic acid of 119 ± 5.21 µM, respectively (Figure 2b). As heptadecanoic acid exerted higher cytotoxicity in normal mammary epithelial cells (MCF-10A) compared to pentadecanoic acid (Figure 2c), pentadecanoic acid was considered for further experiments in the present study. In addition, pentadecanoic acid exerted time-dependent cytotoxic effects in MCF-7/SC at 24 h and 48 h post incubation, with IC_50_ values of 155.5 ± 9.55 µM and 119 ± 5.21 µM, respectively (Figure 2d).

### 3.3. Pentadecanoic Acid Inhibited Migration and Invasion of MCF-7/SC

**Figure 3:**
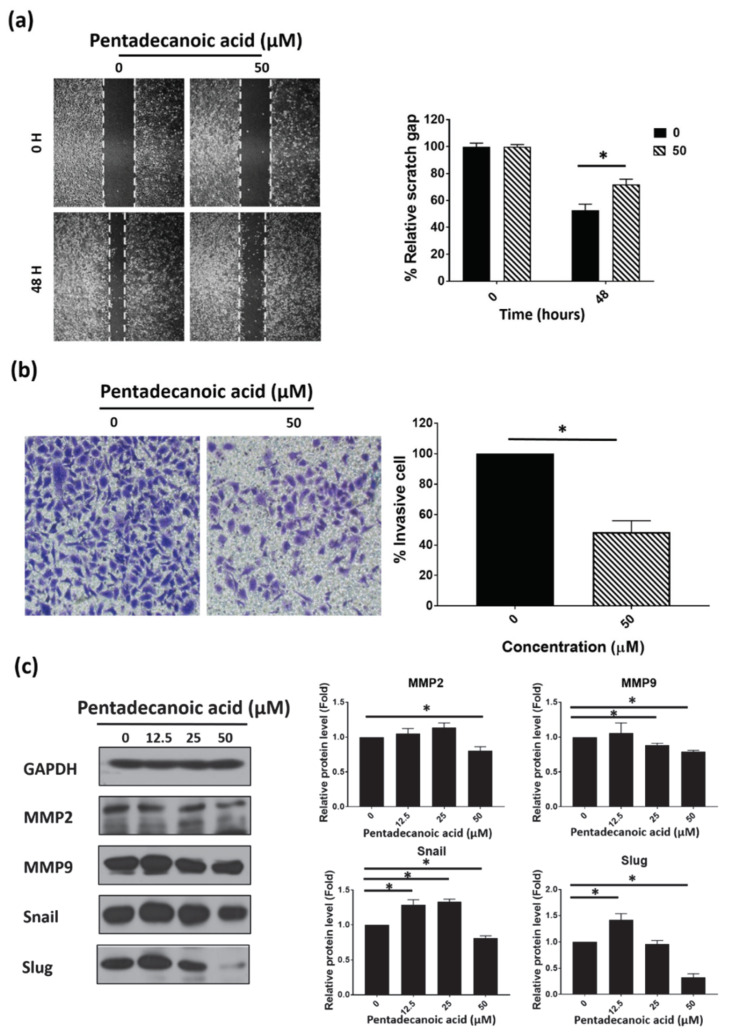
Pentadecanoic acid inhibits the migration and invasion of MCF-7/SC. (**a**) Cell migration was determined by the wound healing assay following 48 h of exposure. (**b**) Invasive cells were stained with crystal violet after treatment with pentadecanoic acid for 48 h (magnification 100×). (**c**) Western blot analysis of epithelial–mesenchymal transition (EMT) markers in MCF-7/SC was performed after pentadecanoic acid treatment for 48 h. GAPDH was used as a loading control. Data are shown as the mean ± standard deviation of three biologically independent experiments. * *p* < 0.05 vs. control.

We next confirmed the effects of pentadecanoic acid on MCF-7/SC migration and invasion using wound healing and trans-well invasion assays. The results of the wound healing and trans-well migration assays demonstrated that pentadecanoic acid could significantly suppress MCF-7/SC migration (Figure 3a) and invasion (Figure 3b) at non-lethal concentrations compared with untreated cells. Then, the effects of pentadecanoic acid on the expression of EMT-associated proteins were examined by Western blot experiments. Matrix metalloproteinase 2 (MMP2), matrix metalloproteinase 9 (MMP9), snail, and slug are closely related to the migration and invasion of a range of human cancer cells. As illustrated in Figure 3c, pentadecanoic acid remarkably suppressed the expression of MMP2, MMP9, snail, and slug dose-dependently. Collectively, these results indicate that pentadecanoic acid could inhibit the migration and invasion of MCF-7/SC.

### 3.4. Pentadecanoic Acid Suppressed the Stemness of MCF-7/SC

**Figure 4:**
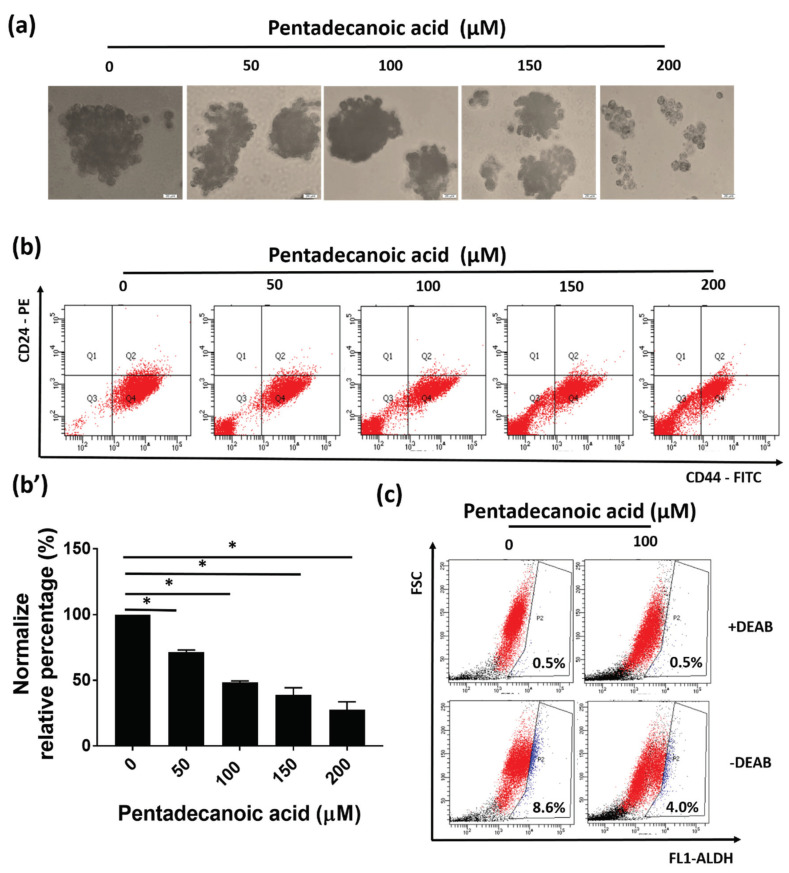

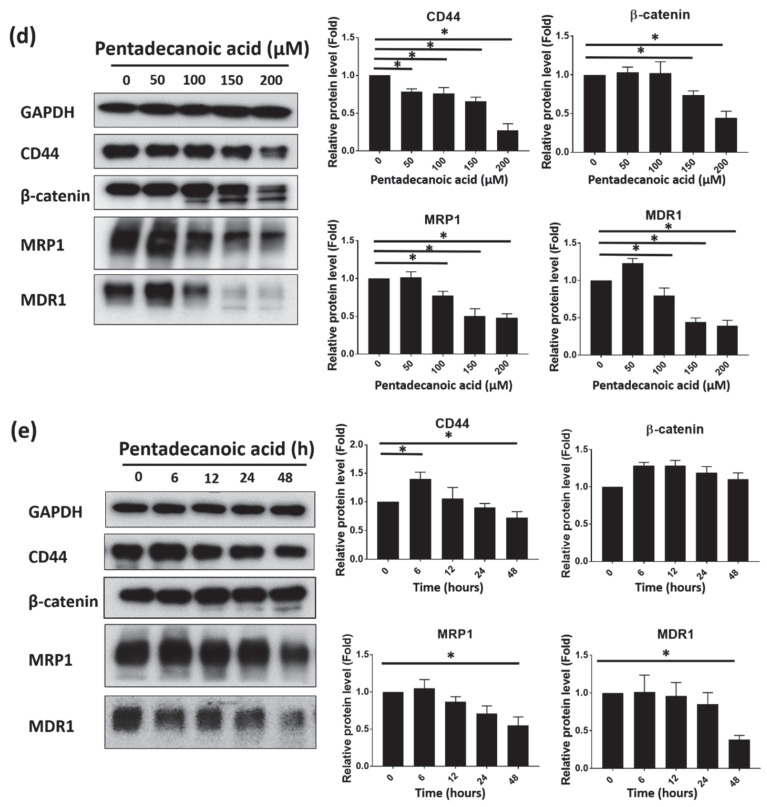
Pentadecanoic acid suppresses the stem like-cell characteristics of MCF-7/SC. (**a**) Effects of pentadecanoic acid on MCF-7/SC mammosphere formation (magnification 100×). (**b**,**b’**) The effects of pentadecanoic acid on the expression of cell surface markers in MCF-7/SC were analyzed by FACS analysis following pentadecanoic acid exposure. (**c**) Flow cytometry analysis of the ALDH^+^ population using the ALDEFLUOR assay kit. Cells were treated with pentadecanoic acid for 48 h prior to the assay. (**d**) The levels of cancer stem cell (CSC) markers were assessed by Western blot experiments following pentadecanoic acid treatment for 48 h. (**e**) The levels of CSC markers as assessed by Western blot experiments following 100 μM pentadecanoic acid exposure. GAPDH was used as a loading control. Data are shown as the mean ± standard deviation of three biologically independent experiments. * *p* < 0.05 vs. control.

As pentadecanoic acid exerted enhanced cytotoxicity in MCF-7/SC compared to in the MCF-7 cells (Figure 2b), we evaluated whether pentadecanoic acid could suppress the stemness of MCF-7/SC. As shown in Figure 4a, pentadecanoic acid significantly reduced the formation of mammospheres in MCF-7/SC in a dose-dependent manner. In addition, the percentage of the CD44^+^/CD24^−^ cell population declined after pentadecanoic acid treatment (Figure 4b). A reduced ALDH activity was also noted following pentadecanoic acid treatment as illustrated in Figure 4c. These observations were further confirmed by Western blot analysis. The expression of the stem cell markers CD44, β-catenin, MRP1, and MDR1 was significantly diminished by pentadecanoic acid treatment in a dose (Figure 4e)- and time (Figure 4f)-dependent manner. Overall, the results demonstrate that pentadecanoic acid can target the stemness of MCF-7/SC time- and dose-dependently. 

### 3.5. Pentadecanoic Acid Suppressd JAK2/STAT3 Signaling in MCF-7/SC

**Figure 5:**
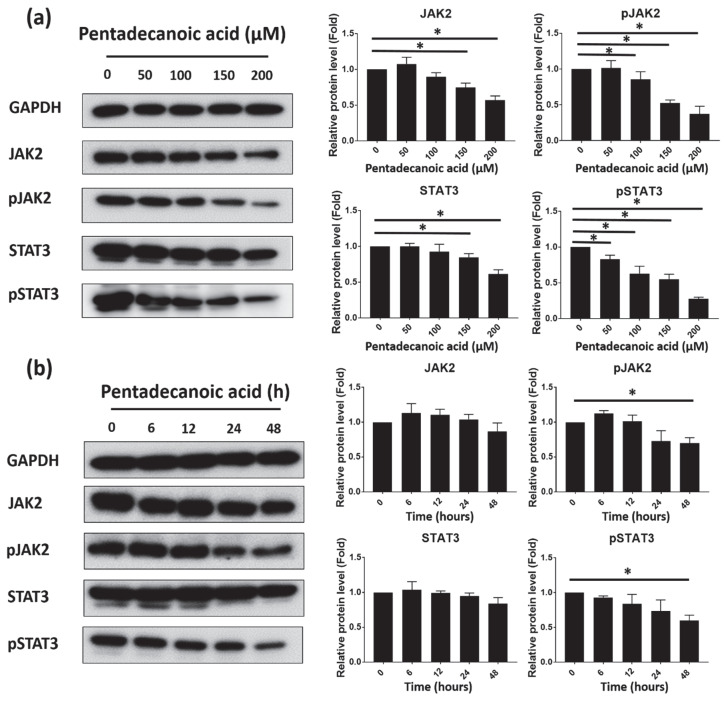

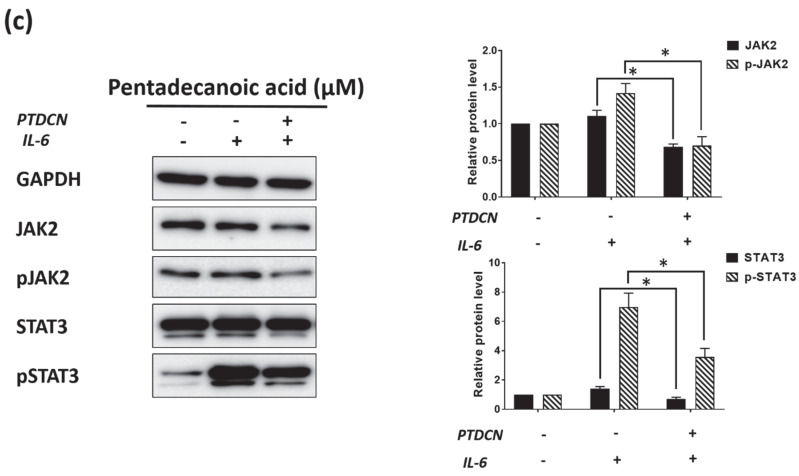
Pentadecanoic acid suppressed JAK2/STAT3 signaling in MCF-7/SC. (**a**) Representative Western blot analysis showing the dose-dependent effects of pentadecanoic acid on the expression of STAT3, pSTAT3, JAK2, and pJAK2 in MCF-7/SC following 48 h of incubation. (**b**) Representative Western blot analysis showing the time-dependent effects of 100 μM pentadecanoic acid on the expression of STAT3, pSTAT3, JAK2, and pJAK2 in MCF-7/SC. (**c**) Effects of pentadecanoic acid on IL-6-inducible JAK2/STAT3 signaling. MCF-7/SC were pre-treated with 150 μM pentadecanoic acid for 48 h and stimulated with 20 ng/mL of IL-6 for 15 min. In Figure 5c, PTDCN indicates pentadecanoic acid. GAPDH was used as a loading control. Data are shown as the mean ± standard deviation of three biologically independent experiments. * *p* < 0.05 vs. control.

The transcriptional activator STAT3 regulates several oncogenes in a range of human cancers [40]. Recent studies have demonstrated the role of JAK2/STAT3 signaling in CSC proliferation and survival. The activation of this pathway has been reported to be involved in the progression, proliferation, apoptosis, metastasis, and chemoresistance of CSCs [15]. After assessing the effects of pentadecanoic acid on the stemness of MCF-7/SC, we investigated whether pentadecanoic acid could suppress JAK2/STAT3 signaling in MCF-7/SC by Western blot analysis. As shown in Figure 5a, b, pentadecanoic acid reduced the expression of total and phosphorylated forms of JAK2 and STAT3 in a dose- and time-dependent manner. However, compared to the total forms, a dramatic reduction in the phosphorylated forms of JAK2 and STAT3 was observed in MCF-7/SC, suggesting that pentadecanoic acid can attenuate the JAK2/STAT3 signaling pathway in MCF-7/SC. According to previous studies, interleukin-6 (IL-6) can activate JAK2/STAT3 signaling, and repression of the IL-6/JAK2/STAT3 signaling axis has been reported to inhibit the migration, invasion, and tumor formation of breast cancer cells [41,42,43]. To further explore the underlying molecular mechanisms, we examined whether pentadecanoic acid can repress IL-6-induced JAK2/STAT3 signaling in MCF-7/SC. As shown in Figure 5c, IL-6 treatment significantly induced the phosphorylation of JAK2 and STAT3 in MCF-7/SC. Interestingly, the results demonstrated that pentadecanoic acid can prevent the IL-6-stimulated phosphorylation of JAK2 and STAT3, highlighting a new function of pentadecanoic acid as an inhibitor of the IL-6/JAK2/STAT3 signaling axis.

### 3.6. Pentadecanoic Acid Induced Apoptosis in MCF-7/SC

**Figure 6:**
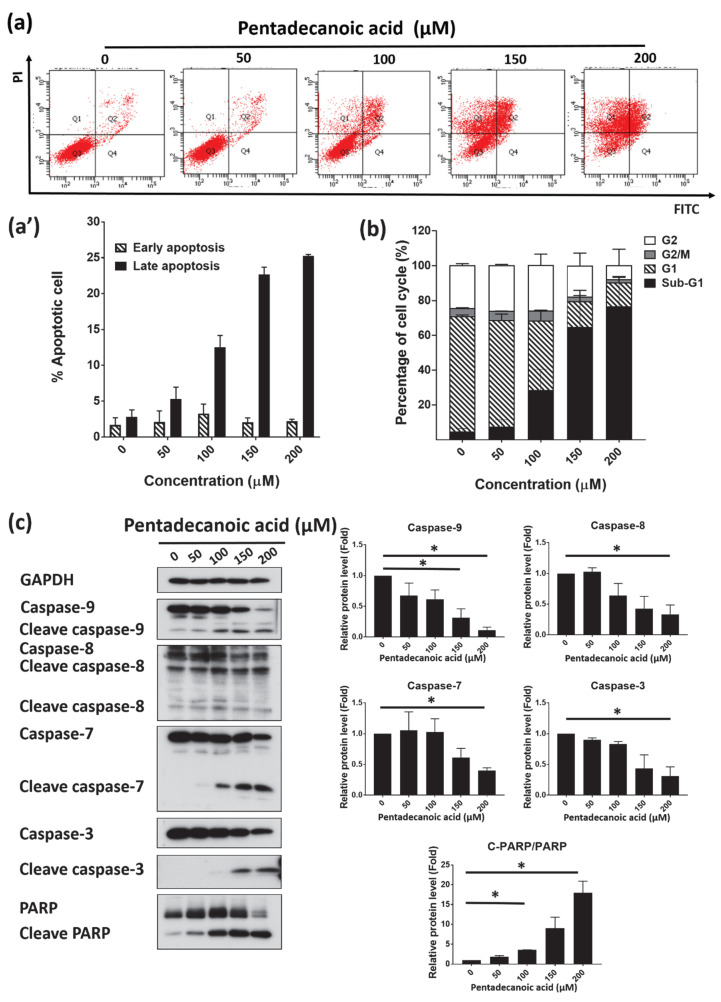
Pentadecanoic acid induced apoptosis in MCF-7/SC. (**a**,**a’**) Fluorescein-conjugated annexin V (annexin V-FITC) vs. propidium iodide (PI) staining analysis showing apoptosis induction following treatment with pentadecanoic acid for 48 h. (**b**) Cell cycle analysis of MCF-7/SC exposed to pentadecanoic acid for 48 h. (**c**) The levels of apoptosis markers were assessed by Western blot experiments following pentadecanoic acid treatment for 48 h. GAPDH was used as a loading control. Data are shown as the mean ± standard deviation of three biologically independent experiments. * *p* < 0.05 vs. control.

Evading apoptosis is one of the key characteristics of cancer cells [44,45]. A number of natural compounds have been identified as promising apoptosis-inducing agents [46,47,48]. Moreover, related to its role in cellular proliferation, multiple studies have demonstrated that STAT3 signaling is associated with cellular apoptosis [49,50,51]. The suppression of STAT3 signaling has been reported to promote apoptosis in several human cancer cells including breast cancer cells [49,50,52]. Following this observation, we checked whether pentadecanoic acid has the ability to induce apoptosis in MCF-7/SC. First, the induction of apoptosis by pentadecanoic acid was confirmed by annexin V/PI staining. As shown in Figure 6a, pentadecanoic acid-treated MCF-7/SC showed signs of early and late apoptosis. At 200 µM, the percentage of late apoptotic cells increased markedly, by 7.25 times compared to the control group. As previous studies have demonstrated that the induction of apoptosis is associated with cell cycle arrest in cancer cells [53], the effects of pentadecanoic acid on the MCF-7/SC cell cycle were evaluated. As shown in Figure 6b, pentadecanoic acid arrested the MCF-7/SC cell cycle at the sub-G1 phase. The sub-G1 population increased from 5.74% to 84.60% (14.74 folds ± 2.43), indicating the induction of apoptosis by pentadecanoic acid through cell cycle arrest in MCF-7/SC. These results were further strengthened by Western blot analysis, which indicated a decrease in the expression of apoptosis markers such as caspase-3, caspase-7, caspase-8, caspase-9, and PARP and an increase in the expression of their cleaved forms (Figure 6c).

## 4. Discussion

Multiple components such as transformed cancer cells, supportive cells, tumor-infiltrating cells, and CSCs in a breast tumor contribute to tumor heterogeneity [54]. Among these, CSCs are resistant to chemo- and radio-therapies and possess unique stem cell properties such as self-renewal and differentiation [55]. Therefore, the development of new treatment strategies that can selectively target cancer stem cells will help to achieve efficient breast cancer treatments. In recent decades, fatty acids have become a novel concern in cancer biology [56,57,58,59,60]. However, the pre-clinical and clinical efficacies of these agents in cancer treatments have not been completely assessed.

Some fatty acids have demonstrated contradictory pre-clinical findings in some in vitro models. For example, palmitic acid (C16:0), an even-chain saturated fatty acid, has been reported to enhance the metastatic potential of melanoma, breast cancer [61], and prostate cancer cells [62] in vitro. Furthermore, palmitic acid has been reported to promote colorectal tumorigenesis in vivo [63]. By contrast, stearic acid (C18:0), another example of an even-chain saturated fatty acid, was shown to inhibit breast cancer cell proliferation in vitro [64,65] and breast tumorigenesis in vivo [66,67], highlighting a dual role of saturated fatty acids in cancer cells. Therefore, it is meaningful to explore the underlying molecular mechanisms of action of saturated fatty acids in human cancer cells. Interestingly, a recent study demonstrated that the OCFA heptadecanoic acid can inhibit the proliferation of chemotherapy-resistant lung cancer cells, indicating the efficacy of OCFAs to target human lung cancer cells [30]. Nevertheless, no studies report the anti-cancer efficacy of odd-chain fatty acids in breast or breast cancer stem cells. Therefore, the lack of investigations assessing the effects of OCFAs in breast cancer cells led us to explore the potential anti-cancer effects of pentadecanoic acid, an OCFA, in a human breast carcinoma stem-like cell line MCF-7/SC established from MCF-7 breast cancer cells. Our study demonstrated that pentadecanoic acid can exert cytotoxic effects in MCF-7/SC with less cytotoxicity to normal breast epithelial cells (MCF-10A) (Figure 2c). Interestingly, compared with the cytotoxic effects in MCF-7 breast cancer cells, the cytotoxic effects of pentadecanoic acid are more specific to MCF-7/SC (Figure 2b), indicating that pentadecanoic acid might play a selective inhibitory role in BCSCs.

In breast cancer patients, a positive correlation has been reported between the CSC population and poor prognosis [68]. Additionally, growing evidence indicates an association between CSCs and ATP binding cassette (ABC) drug transporters in cancer therapies [6,69]. Among the protective mechanisms implicated in CSCs, the overexpression of ABC protein family drug transporters such as MRP1 and MDR1 plays an important role [70]. In this study, we demonstrated that the breast cancer stem-like cells, MCF-7/SC, possess prominent stem cell properties such as an enhanced CD44^+^/CD24^−^ population, reduced ROS levels, and increased mammosphere formation ability compared with the parental MCF-7 cells (Figure 1a–e). Furthermore, MCF-7/SC exhibited higher expression of CD44, MDR1, and MRP1, some known CSC markers, compared to MCF-7 cells (Figure 1d). To identify the effects of pentadecanoic acid on the stemness of MCF-7/SC, we analyzed the CD44^+^/CD24^−^ population, ALDH activity, and mammosphere formation following exposure to pentadecanoic acid. As shown in Figure 4a–c, pentadecanoic acid treatment significantly reduced the CD44+/CD24^−^ population, ALDH activity, and mammosphere formation in MCF-7/SC dose-dependently. As a typical CSC marker, CD44 plays an important role in the self-renewal, angiogenesis, cell motility, and invasion of CSCs [71]. Pentadecanoic acid treatment also reduced the expression of CD44, β-catenin, MDR1, and MRP1 in a dose- and time-dependent manner (Figure 4d,e). It has been identified that targeting the stemness in a tumor is a promising and essential therapeutic strategy for developing novel cancer treatments [72,73].

EMT is considered as a characteristic of CSCs [74]. According to Guen et al. a stem cell population isolated from breast cancer cells exerted elevated expression of EMT markers [75]. In EMT, epithelial cells lose apical–basal polarity and cell–cell adhesion, leading to movement to further sites [76]. This process is necessary for tumor progression [77,78]. Several studies have proven a direct association between EMT and the generation and maintenance of CSCs [79,80]. Matrix metalloproteinases (MMPs)—MMP2 and MMP9, snail, and slug—have widely been used as EMT markers. Our results demonstrated that pentadecanoic acid can significantly inhibit migration and invasion (Figure 3a,b) and the expression of MMP2, MMP9, snail, and slug in MCF-7/SC (Figure 3c), suggesting that pentadecanoic acid could inhibit cell invasion and migration through the suppression of MMPs, snail, and slug.

JAK2/STAT3 signaling has been reported to be responsible for the maintenance of CD44+/CD24^−^ stem like-cell populations isolated from human breast cancer cells [15]. Therefore, the targeting JAK2/STAT3 signaling is considered as a promising therapeutic strategy. Growing experimental evidence demonstrates that Signal Transducer and Activator of Transcription 3 (STAT3) is constitutively activated in a range of human cancers including breast cancer [81]. As an essential component, STAT3 participates in cell proliferation, apoptosis, metastasis, and EMT in breast cancer cells [82,83,84,85,86]. Activated Janus kinase family of kinases (JAKs) activate STAT3 on the Tyr-705 residue at its C-terminus [87]. Activated STAT3 dimerizes and translocates into the nucleus. In the nucleus, activated STAT3 binds to the interferon-gamma activated sequence (GAS) of promoters to regulate the transcription of some of its target genes such as the *Bcl-2* family, *CycinD1*, *c-Myc*, *Survivin*, *MMP2*, and *MMP9* [85,88,89,90]. Yang et al. provided direct evidence that un-phosphorylated STAT3 (U-STAT3) can bind to GAS sequences as a dimer or monomer, implying that U-STAT3 can also play an essential role in the regulation of gene expression [91,92]. In the present study, we observed that pentadecanoic acid can significantly inhibit JAK2/STAT3 signaling in a dose- and time-dependent manner (Figure 5a,b), suggesting that pentadecanoic acid can play a prominent role as an inhibitor of the JAK2/STAT3 signaling pathway in BCSCs. Moreover, to validate the suppression of the JAK2/STAT3 signaling pathway caused by pentadecanoic acid, MCF-7/SC were pre-treated with pentadecanoic acid following IL-6 exposure for 15 min. IL-6, a pro-inflammatory cytokine, is a common upstream activator of the JAK2/STAT3 signaling pathway [93]. Consistent with the previous reports describing that STAT3 can be phosphorylated by the IL-6-stimulated JAK2 [94,95], our results show that IL-6 exposure significantly induced pJAK2 and pSTAT3 expression in MCF-7/SC. Moreover, pentadecanoic exposure dramatically suppressed IL-6-stimulated JAK2/STAT3 signaling (Figure 5c), presenting a novel function of pentadecanoic acid as an inhibitor of the IL-6/JAK2/STAT3 signaling pathway in MCF-7/SC.

Apoptosis is a well-known programmed cell death mechanism associated with the continuous activation of caspases [96]. Caspases can be divided into two classes: the initiator caspases (i.e., caspase-2, -8, -9, and -10) and the effector caspases (i.e., caspase-3, -6, and -7) [96]. In the extrinsic apoptotic pathway, caspase-8 is usually activated by death receptors, whereas in the intrinsic apoptotic pathway, caspase-9 is activated through the apoptosome [97]. Among the effector caspases, the activation of caspase-3 is responsible for the cleavage of other substrates associated with apoptosis (for example, the cleavage of PARP-1) [98]. According to the Western blot experiment results (Figure 6c), pentadecanoic acid exposure resulted in an increase in the expression of cleaved caspase-3, -7, -8, and -9 in MCF-7/SC, indicating that pentadecanoic acid can induce both the extrinsic and intrinsic apoptosis pathways. Several studies have shown that fatty acids can induce apoptosis in a range of human cancers including breast [57,65], prostate [99], and lung [100] cancers. However, these studies have not been able to elucidate the complete molecular mechanisms involved in the induction of apoptosis. By contrast, our study reports for the first time that pentadecanoic acid, one of the OCFAs, can suppress the stemness of MCF-7/SC and promote apoptosis through the inhibition of the JAK2/STAT3 signaling pathway.

## 5. Conclusions

In conclusion, we demonstrate, for the first time, that pentadecanoic acid can exert cytotoxic effects in MCF-7/SC with less cytotoxicity to MCF-10A normal mammary epithelial cells. Furthermore, pentadecanoic acid suppressed the stemness of MCF-7/SC through targeting the JAK2/STAT3 signaling pathway. In addition, pentadecanoic acid induced cell cycle arrest and apoptosis in MCF-7/SC. This study proposes the use of pentadecanoic acid as a novel JAK2/STAT3 inhibitor in breast cancer therapy.

## Abbreviations

ALDH	Aldehyde dehydrogenases
BCSCs	Breast cancer stem cells
DEAB	Dimethylaminobenzaldehyde
DMEM	Dulbecco’s modified Eagle’s medium
DMSO	Dimethyl sulfoxide
EMT	Epithelial–mesenchymal transition
ER	Estrogen receptor
FACS	Fluorescence-activated cell sorting
FBS	Fetal bovine serum
GAS	Gamma activated sequence
H2DCFDA	2′,7′-dichlorofluorescein diacetate
IC_50_	Inhibitory concentration of 50
IgG	Immunoglobulin G
IL-6	Interleukin-6
JAKs	Janus kinase family of kinases
MDR1	Multidrug-resistance protein 1
MMP	Matrix metalloproteinase
MRP1	Multidrug resistance-associated protein 1
MTT	3-(4,5-dimethylthiazol-2-yl)-2,5-diphenyl-tetrazolium bromide
OCFAs	Odd-chain fatty acids
PBS	Phosphate buffered saline
P-gp	P-glycoprotein
PI	Propidium iodide
RIPA	Radioimmunoprecipitation assay
ROS	Reactive oxygen species
RPMI	Roswell Park Memorial Institute
SP	Side population
STAT3	Signal transducer and activator of transcription 3
U-STAT3	Un-phosphorylated STAT3
